# Therapeutic Efficacy of Fuzheng-Huayu Tablet Based Traditional Chinese Medicine Syndrome Differentiation on Hepatitis-B-Caused Cirrhosis: A Multicenter Double-Blind Randomized Controlled Trail

**DOI:** 10.1155/2013/709305

**Published:** 2013-03-06

**Authors:** Ya-Nan Song, Ji-Jia Sun, Yi-Yu Lu, Lie-Ming Xu, Yue-Qiu Gao, Wei Zhang, Xiao-Su Wang, Dong-Ying Xue, Qing-Shan Zheng, Shi-Bing Su

**Affiliations:** ^1^Research Center for TCM Complexity System, Shanghai University of TCM, Shanghai 201203, China; ^2^Shanghai Shuguang Hospital, Shanghai 200021, China; ^3^Shanghai Longhua Hospital, Shanghai University of TCM, Shanghai 201203, China; ^4^Shanghai Yueyang Hospital, Shanghai 200437, China; ^5^Shanghai Putuo Hospital, Shanghai 200060, China; ^6^Center for Drug Clincal Reserch, Shanghai University of TCM, Shanghai 201203, China

## Abstract

*Aim*. To evaluate and predict the therapeutic efficacy of Fuzheng-Huayu tablet (FZHY) based traditional Chinese Medicine (TCM) syndrome differentiation or TCM symptoms on chronic hepatitis B caused cirrhosis (HBC). *Methods.* The trial was designed according to CONSORT statement. It was a multi-center, double-blind, randomized, placebo-controlled trail. Several clinical parameters, Child-Pugh classification and TCM symptoms were detected and evaluated. The FZHY efficacy was predicted by an established Bayes forecasting method following the Bayes classification model. *Results. *The levels of HA and TCM syndrome score in FZHY group were significantly decreased (*P* < 0.05) compared to placebo group, respectively. The efficacy of FZHY on TCM syndrome score in HBC patients with some TCM syndromes was better. In TCM syndrome score evaluation, there were 53 effective and 22 invalid in FZHY group. TCM symptoms predicted FZHY efficacy on HBC were close to Child-Pugh score prediction. *Conclusion.* FZHY decreases the levels of HA and TCM syndrome scores, improves the life quality of HBC patients. Moreover, there were different therapeutic efficacies among different TCM syndromes, indicating that accurate TCM syndrome differentiation might guide the better TCM treatment. Furthermore, the FZHY efficacy was able to predict by Bayes forecasting method through the alteration of TCM symptoms.

## 1. Introduction

Hepatitis B virus (HBV) infection is a major health problem in China. It is one of the important reasons for virus-related liver diseases, such as chronic hepatitis B (CHB), liver cirrhosis (LC), and hepatocellular carcinoma (HCC) [1]. In worldwide, there are 350 million HBV-infected people who have 15–25% risk of dying from the HBV-caused LC or HCC [2]. The five-year survival rate of patients with severe CHB and caused cirrhosis is about 50% [3]. Clinically it lacks the effective drugs for the therapy of hepatitis-B-caused cirrhosis (HBC) so far. 

Fuzheng-Huayu tablet (FZHY), a Chinese herbal medicine formula, affected liver fibrosis in clinical trials [4–6] and animal experiments [7–9]. In the clinical practices of traditional Chinese medicine (TCM), the efficacy of FZHY is based on TCM syndrome, which is the characteristic in body tired and weak, no appetite, face pale white or dark sluggish, tongue dark, or bruises in HBC patients. Moreover, FZHY was able to halt the progress of liver fibrosis through inhibiting the activation of hepatic satellite cells in animal model [8]. However, it has less clinical efficacy evaluation on HBC.

The clinical efficacy evaluation in traditional Chinese medicine (TCM) is based on TCM syndrome, which also known as ZHENG or TCM pattern, is a characteristic profile in clinical symptoms and signs [10]. Therefore, how to collect, analyze and identify the clinical symptoms and signs underlying TCM theory, simultaneously taking example by clinical efficacy evaluation Western medicine is very important to TCM clinical efficacy evaluation. Currently, some Bayes analysis methods have been applied in the clinical efficacy evaluation of herbal medicine and the classification of TCM syndrome, such as Bayesian classification [11], Bayes random-effects estimator method [12], and Bayesian network [13]. Though Bayes forecasting method has been established to use in the individual treatment of chemotherapy [14], it still has been not applied in the clinical efficacy evaluation of TCM. 

In this study, a multicenter, double-blind, randomized, placebo-controlled trail was carried out. The aim was to evaluate and predict FZHY efficacy on HBC with or without TCM syndrome differentiation through the outcome assessment of serum alanine aminotransferase (ALT), aspartate aminotransferase (AST), hyaluronic acid(HA), Child-Pugh score and TCM syndrome score, and the prediction of FZHY efficacy using Bayes forecasting method. 

## 2. Method

### 2.1. Trial Design

The study was designed to a multi-center, double-blind, equal-randomized, placebo-controlled trial and was carried out to evaluate the efficacy of FZHY, reported in accordance to the 2010 CORSORT statements. With intervention allocation ratio 1 : 1, participants randomized to the intervention group would be given FZHY, while the others randomized to the control group would be given placebo.

The study was conducted according to the guidelines of the Declaration of Helsinki and the principles of Good Clinical Practice (China), and obtained the approval of medical ethics committee in Shanghai Shuguang Hospital. The full trial protocol was drawn up and taken care by Center for Drug ClincalReserch of Shanghai University of TCM.

### 2.2. Sample Size

Because of first exploration for FZHY treatment on HBC, there were not any basic parameters provided as references, and we tried to carry out the study with 200 participants and the same participant number in FZHY group and placebo group. In the process of enrollment, 20 participants were excluded considering not meeting inclusion criteria, participating in another study, and some other reasons.

### 2.3. Randomization and Blinding

According to layering subsection random method using DAS statistical software by Center for Drug ClincalReserch of Shanghai University of TCM, random number table was generated on the basis of random proportion. And the table was safely kept by the executive and persons in charge. Placebo treatment group was designed as control group in the study, and placebos were matched to study drugs for taste, color, and size. Drugs and placebos were numbered according to random number table, which was unknown to both doctors and patients. Based on the sequence of participants and the number of drugs and placebos, the interventions were administered.

### 2.4. Participants

The study would include adults if they met the following criteria:

- clinical diagnosis of post-hepatitis B cirrhosis;
- male or female patients between 18~65 years old;
- written informed consent.

The participants would be excluded if they met one of the following criteria:

- Child-Pugh C above 12, or ALT > 2 ∗ ULN, TBiL > 2 ∗ ULN, or combined with hepatic encephalopathy, Obstinate ascites, bleeding tendency, hepatorenal syndrome, or primary liver cancer;
- combine with severe heart, gallbladder, kidney, endocrine system, hemopoietic system, or nervous system disease;
- deformed man by the law;
- pregnancy or breast feeding women, or unwilling to have contraception;
- irritability body constitution, or irritability of the FZHY;
- enter other trials lately one month;
- other cause of cirrhosis.

The assessor (gastroenterologists) was conducted at 4 centers including Shuguang, Longhua, Yueyang, and Putuo hospitals in Shanghai in China (Table 1). And finally 180 participants were included. The clinical information of HBC patients, such as symptoms and signs, was collected from the above 4 hospitals, and then TCM syndromes were classified into Liver stagnation syndrome (LSS), spleen deficiency and damp overabundance syndrome (SDDOS), damp-heat accumulation syndrome (DHAS), liver-kidney Yang deficiency syndrome (LKYDS), blood stagnation syndrome (BSS), and spleen-kidney Yang deficiency syndrome (SKYDS) by 3 senior TCM physicians according to the definition of diagnosis, and TCM syndrome differentiation of liver cirrhosis [15]. Total 45 of clinical symptoms, such as body tired and weak, no appetite, and face pale white or dark sluggish, and 39 of sighs, such as tongue dark or bruises, and pulse float or heavy, were used as TCM symptoms. Each clinical symptom and sign was standard with the degree of clinical manifestation of HBC, which was called TCM syndrome score. The TCM syndrome score was as without, 0; weakness, 1; slight heavy, 2; very heavy, 3, and the signs was as within, 1; without, 2. In order to ensure the repeatability and reliability of TCM syndrome and symptom diagnoses, all patients were diagnosed by 3 senior TCM physicians separately in the same condition, and the final diagnosis was made by a TCM botanic physician. It was bring into the further study when the diagnoses were consistent. 

### 2.5. Interventions

FZHY (SFDA approval no: Z20050546) and placebo (2% FZHY) were prepared and provided by Shanghai Sundise Medicine Technology Development Co., Ltd. (Shanghai, China). There were the same appearance and smell and 0.4 g per tablet in both FZHY and placebo. The quality control and preparing standardization of FZHY was established and enforced according to previous report [16].

180 HBC patients were randomly divided to receive FZHY treatment group (FZHY group, 90 cases) and placebo treatment group (placebo group, 90 cases). The patients were given FZHY or placebo, every day 3 times oral, every time 1.6 g, and a total of taking 6 months.

### 2.6. Outcomes

The study was designed to evaluate the therapeutic efficacy of FZHY and placebo treatment for 6 months on HBC. FZHY efficacy was evaluated as the outcome assessment of ALT, AST, HA, Child-Pugh score, and TCM syndrome sore, and predicted by an established forecasting model based on the Bayes classification method. The Bayes classification method was added after trial commencement. 

The levels of ALT and AST were detected by velocity method, the levels of TBil were detected by endpoint method, and the level of albumin (Alb) was detected by one point method. The detection of PT was carried out by coagulation method. The agents were from STAGO Company (Parsippany, NJ, USA). HA was detected using Hyaluronic Acid Test Kit from Corgenix, Inc. (Westminster, CO, USA).

### 2.7. Statistical Methods

#### 2.7.1. Basic Statistical Analysis

The comparison between treatment groups was evaluated by Student's *t*-test. The level of significance was set at *P* < 0.05.

#### 2.7.2. Efficacy Evaluation

Before and after of FZHY and placebo treated for 6 months, the TCM symptoms, the levels of ALT, AST, HA, Child-Pugh score, and TCM syndrome sore were evaluated. The Child-Pugh scores of HBC patients were recorded and calculated by rating the following five parameters including serum levels of TBil, A1b, PT, ascites, and encephalopathy, and divided into classes A (5-6 points), B (7–9 points), and C (10–15 points) [17, 18]. The efficacy evaluation of TCM syndrome was according to “Guideline for Clinical New Drug Research in Chinese Herbal Medicine” [19]. The standard of TCM syndrome outcome was as follows: TCM syndrome score as without, 0; light, 1; heavy, 2 points. The calculation formula was as follows: the efficacy index of TCM syndrome (*N*) = [(before treatment score − after treatment score)/before treatment score ×100%. The efficacy evaluation standard of TCM syndrome: clinical cure: *N* ≥ 90%; excellent: *N* < 90% ~>60%; effective: *N* ≤ 60% ~>30%; invalid: *N* ≤ 30%. In this study, the Effective was *N* > 30% including above clinical cure, excellent and effective. There were not any changes to trial outcomes after the trial commenced.

#### 2.7.3. Bayes Forecasting Method

Based on the Naïve Bayes classification [20], the Bayes forecasting method was established. It is was required that all symptoms or sighs *x*_1_, *x*_2_, …, *x*_*m*_ are independent and all kinds of classification type *y*_1_, *y*_2_, …, *y*_*n*_ are mutually exclusive complete group. While the event *x*_1_, *x*_2_, …, *x*_*m*_ has appeared, the probability of event *y*_*j*_ was:

(1) P(yj ∣ x1x2⋯xm)  =P(yj)P(x1 ∣ yj)P(x2 ∣ yj)⋯P(xm ∣ yj)∑k=1nP(yk)P(x1 ∣ yk)P(x2 ∣ yk)⋯P(xm ∣ yk).

Among, the *P*(*y*_*j*_) is prior probability; *P*(*x*_*i*_ | *y*_*j*_) is conditional probability, *P*(*y*_*j*_ | *x*_1_ *x*_2_ ⋯ *x*_*m*_) is posteriori probability. Formula (1) is Bayes formula. 

In the classification prediction, firstly based on a lot of reliable history material put forward a training samples, and calculates *n* of *P*(*y*_*j*_) using the constituent ratio of various classification, then calculates *m* × *n* of *P*(*x*_*i*_ | *y*_*j*_). According to the Bayes formula, calculates *n* of *P*(*y*_*j*_ | *x*_1_ *x*_2_ ⋯ *x*_*m*_), which reflecting the possibility of samples belong to the classification.

## 3. Results and Discussion

### 3.1. Participant Flow and Numbers Analysed

**Figure 1:**
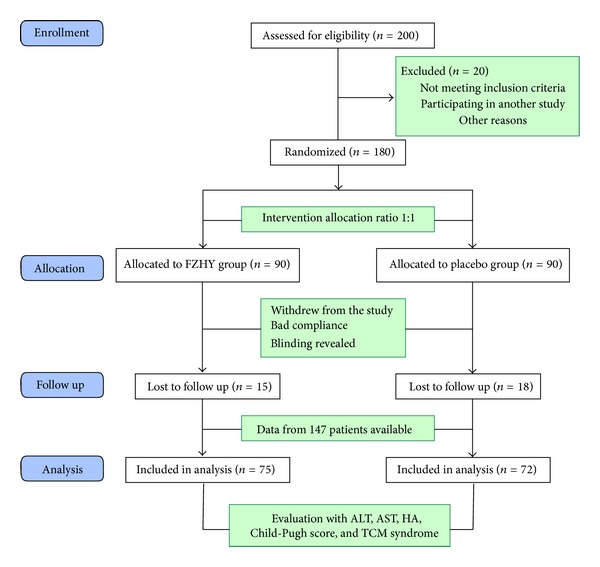
Flow diagram of a multicenter, randomized, controlled, double-blind trial for evaluating therapeutic efficacy of FZHY. The diagram includes information on the excluded participants.

In order to make readers understand our design better, a flow diagram (Figure 1) was used according to the CONSORT (Consolidated Standards of Reporting Trials) statement (http://www.consort-statement.org/). The flow diagram is intended to depict the participants' information from four stages of a trial, including enrollment, intervention allocation, follow-up, and analysis. In this study, the 180 patients included were randomly allocated with the ratio of 1 : 1. In the process of followup, 15 patients in FZHY group and 18 patients in placebo group were lost; thus, data from the other 75 patients in FZHY group and 72 patients in placebo group were available for further analysis. 

### 3.2. Recruitment

Age-eligible HBC patients were recruited from September 2007. Participants enrolled attended clinic visits at the time of randomisation (baseline), one-month interval, three-month interval, and six-month interval. And the study was completed on June 2009.

### 3.3. Baseline Data

All of 180 patients were Chinese yellow race. As shown in Table 2, the ages were from 18 to 65 years. There were 124 male cases (68.89%) and were 56 female cases (31.11%). The numbers of patients with Child-Pugh A, B, and C were 149 (82.78%), 28 (15.56%), and 3 (1.67%), respectively.

### 3.4. Outcomes and Estimation

 To evaluate the therapeutic efficacy of FZHY and placebo treatment for 6 months on HBC, the parameters of liver function such as ALT and AST, liver fibrosis such as HA, and Child-Pugh score were analyzed. Because of the lack of some parameters, 75 cases in FZHY group and 72 cases in placebo group were analyzed. As shown in Table 3, the level of HA in FZHY group was significantly decreased compared to placebo group (*P* < 0.05). The levels of ALT and AST in FZHY group were decreased compared to placebo group, but there were no significant difference between FZHY and placebo groups (*P* > 0.05). Moreover, In the Child-Pugh score, there were no significant changes between both FZHY and placebo groups, and there also were no significant difference between Child-Pugh class A and Child-Pugh classes B and C (data not shown). The *P* values of before and after represented the significance of difference between FZHY group and placebo group before and after treatment, respectively. *P* values of FZHY group and placebo group represented the significance of difference between before treatment and after treatment in FZHY group and placebo group, respectively. 

The formation of liver fibrosis is one of the characteristics of LC. HA is a parameter of liver fibrosis and an independent predictor of LC [21, 22]. Previous studies have reported that HA is an important parameter while FZHY reduced liver fibrosis in clinical trials [4–6] and animal experiments [7–9]. In this study, our results also indicated FZHY may improve liver fibrosis through decreasing the levels of HA in HBC patients.

Furthermore, in 75 cases of FZHY group, there were Child-Pugh score class A, 68 cases; B, 7 cases; C, 0 cases. After FZHY treatment for 6 months, Child-Pugh score class A, 71 cases; B, 4 cases; C, 0 cases. Child-Pugh scores classes were decreased to 22 cases, not changed 43 cases, and increased 10 cases. In 72 cases of placebo group, there were Child-Pugh score class A, 57 cases; B, 13 cases; C, 2 cases. After FZHY treatment for 6 months, Child-Pugh score class A, 53 cases; B, 18 cases; C, 1 cases. Child-Pugh score classes were decreased to 19 cases, not changed 33 cases, and increased 20 cases. 

Child-Pugh classification is the common liver reserve function classification standard of cirrhosis, guides treatment, prognosis, and drug efficacy evaluation, and has very important reference value [17, 18]. Triantos et al. [23] has retrospectively analyzed the clinical data of 1234 patients with liver cirrhosis and found that Child-Pugh classification is significant to predict the long-term survival situation.For the TCM efficacy evaluation, Li et al. [24] have constructed a system of therapeutic efficacy evaluation using Child-Pugh classification. As a parameter of liver compensatory function, Child-Pugh score has been used in the efficacy evaluation of Chinese herbal medicine [25] and combined with chemotherapy [26]. In this study, we found that FZHY can reduce or delay the increase of Child-Pugh score in part of HBC and indicated that the prognosis of these HBC patients might take a turn for the better after FZHY treatment for 6 months, but it was not a significant difference compared to placebo groups. 

Because of the first exploration of FZHY treatment for HBC, the limitation of the trial design without sample size calculation could not be neglected. This study was carried out as a reference for further related studies. The evaluation of FZHY efficacy including Child-Pugh classification evaluation needs much evidence through further clinical trial, with more reasonable sample size and a long-term treatment (more than 12 months). 

### 3.5. Ancillary Analyses

#### 3.5.1. Efficacy of FZHY on TCM Syndromes in HBC

In HBC as a chronic disease, the symptoms of patients are also an important index of efficacy evaluation in TCM. TCM syndrome sore, a semiquantitative evaluation method for symptoms and signs, has been used in the efficacy evaluation in TCM clinical practice [19, 27, 28]. As shown in Table 4, in this study, the levels of TCM syndrome score in FZHY group were significantly decreased, compared to placebo groups (*P* < 0.05). As the TCM syndrome differentiation, the levels of TCM syndrome score were decreased compared to placebo groups (*P* < 0.05) in LSS, SDDOS, DHAS, and LKYDS, respectively. There was no significant difference between FZHY group and placebo groups (*P* > 0.05) in BSS and SKYDS. Moreover, the levels of HA were decreased compared to placebo groups (*P* < 0.05) in LSS. In ALT, AST, HA, and Child-Pugh score, there was no significant difference between FZHY and placebo groups (*P* > 0.05) in other TCM syndromes. Since there was different therapeutic efficacy among different TCM syndromes, it indicated that accurate TCM syndrome differentiation might guide the better TCM treatment.

What is more, in the TCM syndrome score evaluation, there were 53 effective and 22 invalid patients in FZHY group, but 26 effective and 47 invalid patients in placebo group; there was significant difference between FZHY and placebo groups (*P* < 0.01). It was indicated that FZHY improved TCM syndrome score in LSS, SDDOS, DHAS, and LKYDS, which may reflect the global improvement of symptoms and life quality in those HBC patients. 

As a clinical manifestation, the symptoms reflecting feels and mental state of patients usually were rejected in the efficacy evaluation of current drugs. However, because of embodying the holistic situation of patients, the classification and the characteristic finding from symptoms and signs of patients is the basis of diagnosis of diseases and efficacy evaluation or prediction in TCM clinical practice. So we further demonstrated the possibility of efficacy prediction by symptoms and signs of patients compared with Child-Pugh score evaluation. 

#### 3.5.2. Efficacy Prediction of FZHY on Child-Pugh Score by Bayes Forecasting Method

For the Bayes prediction analysis of FZHY efficacy, it took training set 147 cases (FZHY group, 75 cases and placebo group, 72 cases) and test set 20 cases (FZHY and placebo group, each 10 cases). While Child-Pugh score in the grading difference between before and after treatments was as the classification events, the probabilities of FZHY and placebo efficacies were able to indicate as the changed situation of Child-Pugh score classification. As shown in Table 5, according to the testing of training set and test set of Bayes forecasting method, it was predicted that the probability of Child-Pugh score decreased 30% and 10%, not changed 60% and 60%, and increased 10% and 30% in FZHY and placebo groups, respectively. In the efficacy prediction from 84 of TCM symptoms, the probability of Child-Pugh score decreased 20% and 10%, not changed 80% and 70%, and increased 0% and 20% in FZHY and placebo groups, respectively. In the efficacy prediction from clinical parameters such as ALT, AST, PT, and HA, the probability of Child-Pugh score decreased 60% and 12%, not changed 38% and 15%, increased 3% and 73% in FZHY and placebo groups, respectively. It suggested that TCM symptoms predicted FZHY efficacy on HBC were close to Child-Pugh score than that of clinical parameters, using Bayes forecasting method. 

Moreover, the efficacy prediction from 84 of TCM symptoms showed that the posteriori probability of Child-Pugh score decreased 99.99% in FZHY group and increased 98.69% in placebo groups, indicated the possibility of FZHY treated HBC. These predicted results were similar with the statistical results of Child-Pugh score.

Bayesian classification model is a kind of classification model in statistical method. Bayes' theorem is one of the most important formulas in Bayesian theory, and it is the theoretical basis of Bayesian learning method. The Bayesian classification model subtly links up the prior probability and posterior probability of events, using prior information and sample data information to determine events of the posterior probability [20]. Previous studies have established a TCM diagnosis model for the efficacy evaluation of coronary heart disease using Bayesian classification model [11], and also established an empirical Bayes random-effects estimator method, to evaluate efficacy of herbal medicines and antidepressants treatment in depression patients [12]. Additionally, Bayesian network has been used in the efficacy prediction of Chinese medicine components [13]. In this study, we have established Bayes forecasting method following the Bayes classification model and using TCM symptoms predicted effectively FZHY efficacy on Child-Pugh scores of HBC patients. It provides a reference for clinical efficacy evaluation following TCM symptoms, and indicated that the usefulness of symptoms in TCM efficacy evaluation.

However, Bayes methods were confined to analyze the probability of clinical efficacy evaluation events, and have known the prior probability, and then calculate its posterior probability. It limits the application of Bayes methods in the clinical efficacy evaluation. 

## 4. Conclusion

The study was a multicenter, double-blind, randomized, and controlled trial research, which the therapeutic efficacy of FZHY on HBC for 6 months was evaluated as a clinical outcome assessment. FZHY therapy improved HA and TCM syndrome score in HBC. Among, FZHY therapy has better efficacy on HA in LSS, and on TCM syndrome score in LSS, SDDOS, DHAS, and LKYDS than other TCM syndromes. There were not any adverse reactions in patients. Moreover, the therapeutic efficacy of FZHY based on Child-Pugh score can be predicted by TCM symptoms using Bayes forecasting method. The results suggested there is a possibility of FZHY efficacy on HBC, and FZHY treatment-based TCM syndrome differentiation is useful to improve the life quality of HBC patients.

## Figures and Tables

**Table 1:** Allocation information of patients with CHB-caused cirrhosis.

Allocation information	Patients (%)
Research centers	
Shuguang hospital	54 (30)
Longhua hospital	56 (31.11)
Yueyang hospital	30 (16.67)
Putuo hospital	40 (22.22)
Treatment groups	
FZHY	90 (50%)
Placebo	90 (50%)

**Table 2:** Clinical data of patients with CHB-caused cirrhosis.

	Patients (%)
Mean age (yr)	50.91 ± 8.36
Male (%)	124 (68.89)
Female (%)	56 (31.11)
Child-Pugh classification	
A	149 (82.78)
B	28 (15.56)
C	3 (1.67)

**Table 3:** Efficacy of FZHY on ALT, AST, HA, and Child-Pugh score in HBC.

Index	Treatment	FZHY group	Placebo group	*P* value
		(mean ± SD)	(mean ± SD)	
		(*n* = 75)	(*n* = 72)	
ALT (U/L)	Before	49.96 ± 47.29	46.38 ± 33.07	0.5996
	After	39.74 ± 26.48	39.97 ± 29.44	0.9602
	*P* value	0.1088	0.2229	

AST (U/L)	Before	57.69 ± 44.96	58.89 ± 42.92	0.8700
	After	50.53 ± 33.40	47.36 ± 28.94	0.5421
	*P* value	0.2757	0.0614	

HA (ng/mL)	Before	219.88 ± 193.08	194.28 ± 155.21	0.3969
	After	140.35 ± 104.02	171.66 ± 156.99	0.1613
	*P* value	**0.0026**	0.3980	

Child-Pugh (score)	Before	5.45 ± 0.99	5.86 ± 1.43	0.0468
	After	5.46 ± 0.90	5.57 ± 1.22	0.5354
	*P* value	0.9746	0.1939	

**Table 4:** Efficacy of FZHY on TCM syndrome score in HBC.

TCM Syndrome	Treatment	FZHY group	Placebo group	*P* value
		(mean ± SD)	(mean ± SD)	
LSS (*n* = 23)	Before	28.00 ± 9.01	30.91 ± 11.90	0.5135
	After	17.33 ± 7.98	25.00 ± 8.77	**0.0396**
	*P* value	**0.0056**	0.2000	

SDDOS (*n* = 29)	Before	21.05 ± 10.68	19.60 ± 9.83	0.7237
	After	13.63 ± 6.37	14.00 ± 10.54	0.9071
	*P* value	**0.0134**	0.2351	

DHAS (*n* = 29)	Before	27.13 ± 9.79	27.21 ± 15.03	0.9863
	After	15.80 ± 13.29	16.00 ± 14.02	0.9688
	*P* value	**0.0128**	0.0515	

LKYDS (*n* = 28)	Before	24.30 ± 6.63	21.06 ± 8.31	0.2997
	After	9.40 ± 5.74	15.61 ± 7.93	**0.0392**
	*P* value	**<0.0001**	0.0524	

BSS (*n* = 32)	Before	29.53 ± 15.50	37.41 ± 13.44	0.1338
	After	20.07 ± 13.69	24.88 ± 13.44	0.3240
	*P* value	0.0871	0.0105	

SKYDS (*n* = 6)	Before	25.50 ± 5.97	28.50 ± 6.36	0.5989
	After	16.00 ± 6.68	15.00 ± 9.90	0.8868
	*P* value	0.0783	0.2462	

**Table 5:** Prediction probability of Child-Pugh score, TCM symptoms, and clinical parameters in FZHY and placebo groups.

Treatment groups	Child-Pugh score	Prediction probability (%)		
		Child-Pugh score	TCM symptom score	Clinical parameters
FZHY group (*n* = 75)	Decreased	30	20	60
	Not changed	60	80	38
	Increased	10	0	3

Placebo group (*n* = 72)	Decreased	10	10	12
	Not changed	60	70	15
	Increased	30	20	73
